# Reciprocal Interactions Between Regulatory T Cells and Intestinal Epithelial Cells

**DOI:** 10.3389/fimmu.2022.951339

**Published:** 2022-07-04

**Authors:** Zhiqiang Jiang, Chuan Wu

**Affiliations:** ^1^ Sun-Yat Sen University, School of Medicine, Guangzhou, China; ^2^ Experimental Immunology Branch, National Cancer Institute, National Institute of Health (NIH), Bethesda, MD, United States

**Keywords:** regulatory (treg) cell, intestinal epithelia cell, intestinal barrier, microbiota, dietary antigen

## Abstract

It has been well established that Foxp3+ regulatory T cells (Treg cells) play a crucial role for immune repression and tolerance, protecting the body from autoimmunity and inflammation. Previous studies indicate that intestinal Treg cells are one specialized population of Treg cells, distinct from those in other organ compartments, both functionally and phenotypically. Specific external and internal signals, particularly the presence of microbiota, shape these Treg cells to better cooperate with the gut ecosystem, controlling intestinal physiology. The integrity of intestinal epithelial barrier represents a key feature of gut immune tolerance, which can be regulated by multiple factors. Emerging evidence suggests that bidirectional interactions between gut epithelium and resident T cells significantly contribute to intestinal barrier function. Understanding how Treg cells regulate intestinal barrier integrity provides insights into immune tolerance-mediated mucosal homeostasis, which can further illuminate potential therapeutic strategies for treating inflammatory bowel disease and colon cancer.

## Introduction

Regulatory T cells (Treg cells) are a specialized T cell subset which play a critical role in controlling immune homeostasis and peripheral tolerance (1, 2). Intestinal Treg cells mainly develop and differentiate in the thymus as thymic Treg (tTreg) cells, or can be induced in the periphery as peripheral Treg (pTreg) cells (3, 4). tTreg cells are generated after self-antigen recognition by T cell receptor in the thymus while pTreg cells are derived by non-self-antigen from naïve T cells. While these two types of Treg cells show complementary functions and different genetic signatures, they both express master transcription factor Foxp3 (5, 6). The function of Foxp3^+^ Treg cells for gut physiology has been documented in patients with immunodysregulation polyendocrinopathy enteropathy X-linked (IPEX) syndrome who lost the Treg cells (7). These patients exhibit symptoms of spontaneous inflammation in multiple organs, with most severe disorders on the mucosal surfaces, including the gastrointestinal (GI) tract (8). Plus, Foxp3 deficient mice (Scurfy) as well display severe autoimmunity in the gut (5, 9). These findings indicate that Treg cells are crucial for the intestinal immune tolerance. Considering the distinct antigen repertoires, intestinal pTreg cells are mainly responsible for immune tolerance against environmental insults, whereas tTreg cells protect the tissue from autoreactive responses.

The intestinal epithelium represents the largest interface which protects the body from potential danger while sensing external milieu. The monolayer of intestinal epithelial cells (IECs) form a physical barrier to segregate external environment from the intestinal tissues. Given the constant challenges and insults from dietary and microbial antigens, the integrity of intestinal epithelium barrier is a key feature of gut homeostasis (10). In addition to immune suppression, newly emerging evidence suggests that intestinal Treg cells also exert function for epithelium tissue repair and mucosal barrier maintenance (11). Hence, to elucidate how Treg-IEC crosstalk participates in gut physiology and pathophysiology is essential for the comprehension of tissue adaptation of Treg cells in the intestinal microenvironment.

In this Review, we will summarize and discuss the current understanding of how mutualism between Treg cells and IECs contribute to GI physiology and immune tolerance.

## Gut Treg Subsets

In general, tTreg cells infiltrating in lamina propria (LP) inductive site origin from those tTreg cells propagating in peripheral blood, while pTreg cells accumulating in LP inductive site are mainly comprised by locally differentiated naïve T cells (12). The surface homing molecules CCR7 and CD62L direct tTreg and naïve T cells migrate into gut-associated lymphoid tissue (GALT) or gut-draining mesenteric lymph nodes (mLN). In these lymphoid compartments, tTreg cells expand when expose to unknown signals (13) and a substantial proportion of the naïve T cells differentiate into pTreg cells. Thereafter tTreg and pTreg cells migrate into LP effective site facilitated by α4β7 integrin and CCR9 signaling (14, 15). Unlike tTreg cells, pTreg cells expand inside of LP after exposed to commensal and dietary antigens (13). With an exception of the common pTreg homing route, there remain some pTreg cells found in LP, differentiated by TGF-β and retinoic acid (RA) producing eosinophils (16). In concert with freshly infiltrated tTreg and pTreg cells, there is also a subset of memory Treg cells resident in LP expand and exert immune suppression functions when induced in the gut. These CD103 expressing memory Treg cells are generated in a previous induction event and quiescent to exhibit tissue resident feature to join in the Treg pool to maintain mucosal homeostasis (17).

Although tTreg and pTreg cells are both able to exert immunosuppression function in the gut, they function independently and synergistically to maintain mucosal tolerance. tTreg cells and pTreg cells have different TCR repertoires, and thus response to different antigens. tTreg cells normally recognize self-antigen, and therefore response to those exposed antigens expressed by IECs, particularly under certain intestinal perturbations such as sterile injuries (18). pTreg cells normally recognize alien-antigens such as dietary metabolites and microbe-antigens and expand at the induction site (19, 20). In addition, strong TCR affinity facilitates the generation of a small portion of cross-react Treg cells with not fully elucidated reasons (18), including self-antigen responding pTreg cells (21) and foreign antigen responding tTreg cells (22). The variety of TCR repertoires covered by tTreg cells and pTreg cells are both required in regulating intestinal immune responses. It has been shown that adoptive transfer of tTreg cells alone is not sufficient to fully rescue Foxp3 deficiency during murine model of colitis, unless Foxp3^–^CD4^+^ T cells are co-transferred, suggesting that both tTreg and pTreg cells are required for optimal protection during intestinal inflammation (23, 24). These findings shed light on developing Treg transfer therapy for potential treatment of human IBD patients.

## IEC-Mediated Intestinal Treg Cells Induction and Function

### IEC-Expressed MHC-II Independent Intestinal Treg Cells

Different studies have shown that intestinal Treg cells can be controlled *via* both IEC independent and dependent manners. Interaction between IEC and dendritic cell (DC) facilitates generation of tolerogenic DC *via* TGF-β and RA, which promotes intestinal Treg cells differentiation and restrains inflammation of colitis (25). Meanwhile, IECs are known to secret exosomes to the extracellular environment, which induce the tolerogenic properties to DCs for the generation of Treg cells in the gut (26). Additionally, other IEC-derived factors such as cytokines are as well known to modulate Treg cells differentiation and function. For instance, IEC-derived IL-18 modulates effector T cell differentiation in the gut which indirectly influence Treg function (27). Another study indicates that during intestinal tumorigenesis, IECs promotes specific subset of KLRG1^+^GATA3^+^ Treg cells accumulation mediated *via* IL-33 (28).

### IEC-Expressed MHC-II Dependent Intestinal Treg Cells

Complement to the DC studies, intestinal Treg cells can also be directly induced by MHC class II (MHC-II) on IECs. It has been shown that both human and mouse IECs express MHC-II molecules (29–32). IECs single cell survey identifies the expression of MHC-II on IECs (33), suggesting that IECs function as non-conventional APCs (34). The induction of MHC-II on IECs has been demonstrated to be IFN-γ−dependent (35–39). It has been reported that IEC-derived MHC-II is sufficient to induce effector CD4^+^ T cells activation in GvHD model (37). Several studies have implicate that IECs preferentially promote suppressive Treg cell responses (38). Loss of MHC-II on IECs results in elevated levels of colitis associated with reduced Treg cells (34, 38). The expression of antigens by IECs leads to the proliferation of antigen-specific Treg cells in the intestine, which is further shown to be MHC-II-dependent (40). Moreover, intestinal mononuclear phagocytes (MNPs) have been reported to acquire MHC-II from IECs, subsequently assisting the generation of Treg cells (41). However, contradictory data show that MHC-II molecules are dispensable for T cell activation during murine colitis (42), raising the possibility that IEC-mediated T cell activation is context-dependent. Additional to IEC-mediated Treg cell expansion, recent study demonstrates that intestinal Treg cells are converted into CD4^+^Foxp3^–^ IELs to control intestinal inflammation, indicating the critical role of IECs in controlling environmental adaptation of Treg cells in the gut (43). IECs from small intestine also provide a unique IL-2 independent milieu for the maintenance and survival of Treg cells (44). Altogether, the microenvironment of epithelium calibrates cellular and functional properties of Treg cells to cope with dynamic change in the gut.

## Microbiota-Derived Intestinal Treg Cells

IECs are critical for microbial-mediated T cell differentiation and accumulation. It has been long established that segmented filamentous bacteria (SFB) promote intestinal Th17 cell differentiation which requires the direct adhesion of SFB on epithelium (45–47). The SFB-IEC interaction leads to the production of serum amyloid A (SAA) from IECs which is critical for Th17 cell differentiation (48). Loss of such interaction compromises the induction of Th17 cells, indicating IEC plays a role of a key mediator in T cell responses to microbes (48). Similarly, gut Treg cells have also been shown to be induced from naïve T cells by antigens derived from commensal bacteria, which are known as inducible Treg cells (49). It has been reported that commensal bacteria such as Clostridium species and *B. fragilis* are able to induce peripheral Treg cells *via* IEC dependent or independent manners (50, 51). The Clostridia colonize the mucus layer without direct adhesion to IECs. The colonization of Clostridium species is found to impact on IECs for the production of TGF-β and indoleamine 2,3-dioxygenase (IDO), which could contribute to the induction of colonic Treg cells (52, 53). More importantly, *de novo* generation of intestinal Treg cells may require synergistic effects with different Clostridia species, given the fact that a single species is insufficient in polarizing Treg cells (54). Additional to TGF-β and IDO-derived from IECs, Clostridia may also induce Treg cell generation *via* producing short chain fatty acids (SCFAs) by diffusing through the epithelium to LP (55–58). Moreover, gut bacteria also generate secondary bile acids which can modulate the balance of Th17 and pTreg cells for intestinal immune homeostasis. (59). While Clostridia species are known to regulate Treg cells *via* IEC dependent manners, other microbiota species including *Lactobacilli* and *Bifidobacteria*, can also induce and activate colonic Treg cells by IEC independent manners (50, 60–62). It is now commonly recognized that microbiota modulates T cell differentiation and function in the gut for intestinal physiology (63). Given that Clostridia and Bacteroides species are two prominent members of the mammalian gut microbiota, such microbiota-mediated Treg cell regulation could be one machinery for the maintenance of gut homeostasis. Recent study further elucidates that mucosa-associated fungi also modulates gut Th17 responses for intestinal barrier function (64). Specifically, both *Candida albicans* and *Staphylococcus aureus* are identified to be strong inducers of human Th17 responses (65, 66). These findings implicate that T cell differentiation and function could be regulated by a diverse community of bacteria, viruses, protozoa, and fungi within the GI tract (67). Given close proximity of gut microbiota and epithelium barrier, IECs play a critical role in bridging the crosstalk between different microbes and hosts for immune regulation in the gut. The precise mechanisms of how IECs collaborate with different microbial for immune tolerance is still under investigation, including Treg cells generation and function.

## Diet-Derived Intestinal Treg Cells

Dietary components largely influence the development and function of intestinal Treg cells, which can be mediated by IEC-dependent and -independent manners. Dietary antigens are known to directly induce RORγt^+^ pTreg cells which are essential for the induction of oral tolerance (19, 68). Dietary vitamin A-derived retinoic acid regulates the differentiation and accumulation of Treg cells, which exerts both pro- and anti-inflammatory functions (69, 70). The metabolite of vitamin D3, 1,25-dihyroxyvitamin D3 can promote Treg cell differentiation (71). Interaction between vitamin B9 and its receptor (folic acid receptor 4) on Treg cells facilitates colonic Treg cell survival (72), protecting the mice from colitis (73). Additionally, vitamin C transporter was found to highly expressed on Treg cells. Vitamin C treatment leads to impaired suppressive function of tTreg cells, whereas it promotes pTreg cell generation both *in vitro* and *in vivo* (74). High salt diet (HSD) has also been reported to promote pathogenic Th17 responses *via* SGK1-Foxo1 signaling pathway while dampening Treg cell function, enhancing the susceptibility of autoimmunity and inflammation (75–78). Moreover, it has been suggested that HSD modulates gut microbial responses for proinflammatory T cells generation in human (79). And clinical study shows that dietary sodium intake positively correlates with the severity of autoimmunity (80). Further, another study demonstrates that dietary-derived sugar, D-mannose, induces Treg cell generation in both human and mouse cells by promoting TGF-β activation. Supplement of D-mannose represses proinflammatory responses in animal models of autoimmunity (81). Moreover, dietary fibers can be fermented and converted into SCFAs through gut microbiota. Various studies suggest that SCFAs stimulate Treg cell differentiation, expansion and accumulation through activation of different G protein-coupled receptors such as GPR43 (58), GPR109A (82) and GPR15 (83). Tryptophan is another critical food component as an essential amino acid. It can be metabolized to kynurenin through IECs which modulates Treg cell development (84). Tryptophan is also the precursor of vitamin B3. Vitamin B3 binds to its receptor GPR109A on macrophages and DCs in the gut, leading to differentiation of Treg cells. Loss of GPR109A results in elevated levels of intestinal inflammation (82). Collectively, these pieces of data indicate that both dietary components modulate Treg cell generation and function in the gut, providing insight of dietary-based therapies in controlling intestinal inflammation.

## Treg Cells Modulate Intestinal Epithelium Barrier Functions

Foxp3^+^ Treg cells play a critical role in regulating IEC homeostasis and intestinal barrier integrity. Although various of cellular sources contribute to intestinal IL-10, as one major effector molecule from Treg cells, Treg cell-derived IL-10 has been demonstrated to play a key role for the maintenance of mucosal immune homeostasis (85, 86). Recent study indicates that Treg cells are required for intestinal stem cells (ISC) renewal *via* IL-10. Loss of Treg cells results in decreased ISC frequency with elevated levels of IEC differentiation (35). T cell-derived IL-10 has been reported to regulate the IECs function *via* inhibiting their fucosylation (87). Further, IL-10 is demonstrated to suppress Fas-mediated IEC apoptosis (88), and protects IEC from endoplasmic reticulum stress for epithelium barrier integrity (89, 90). Given the immune regulatory function of Treg cells in the gut, they are thus able to control epithelium barrier function indirectly by impacting other immune cells. For instance, it is known that Treg cells control the abundance of Th17 cells in the gut. And intestinal Th17 cells-derived cytokines such as IL-17 and IL-22, are beneficial for mucosal barrier function (91–93). Moreover, a previous study indicated that Treg cells improve intestinal barrier function by regulating neutrophil infiltration during heatstroke (94). Treg cells have also been shown to enhance intestinal barrier function by repressing type 2 responses during food allergy (95). The process of generating pTreg cells from naïve T cells carrying environmental antigen specific TCRs is important since it can prevent these T cells from eliciting harmful immune responses. pTreg cell deficient mice exhibit spontaneous inflammation in the GI tract associated with altered microbiota (96). Hence, the reciprocal interactions between IEC and Treg cells are delicately balanced by the gut microenvironment while controlling intestinal barrier physiology.

## Conclusions

Intestinal Treg cells are critical for establishing gut tolerance and host defense. The heterogenicity of these Treg cells are beneficial for protecting the intestinal tissue from various sources of insults. Importantly, the IECs play a key role in connecting environmental cues to tissue immune system for the induction, expansion and function of Treg cells. While the role of IEC as non-canonical APC has been studied, further investigation is still required to illustrate the molecular mechanism of IEC-Treg cell crosstalk. These include correlation of spatial expression pattern of MHC-II on IECs with Treg cell distribution, intracellular signaling pathways of antigen process and presentation by IECs and how specific mediators produced by IECs mediate Treg cells generation and function. Moreover, because of the heterogeneity of IEC population, it will be essential to interrogate in detail that whether and how Treg cells regulate different enterocyte subsets for mucosal neuroendocrinal responses beyond intestinal barrier function. The understanding of the cellular and molecular mechanisms responsible for reciprocal regulation between Treg cells and IECs could provide new insights into how Treg cells control tissue homeostasis on different barrier surfaces for development of therapeutic interventions.

## Author Contributions

CW and ZJ wrote the manuscript. All authors contributed to the article and approved the submitted version.

## Funding

This work was supported by the Intramural Research Program of the NCI, NIH, United States.

## Conflict of Interest

The authors declare that the research was conducted in the absence of any commercial or financial relationships that could be construed as a potential conflict of interest.

## Publisher’s Note

All claims expressed in this article are solely those of the authors and do not necessarily represent those of their affiliated organizations, or those of the publisher, the editors and the reviewers. Any product that may be evaluated in this article, or claim that may be made by its manufacturer, is not guaranteed or endorsed by the publisher.
